# Bitemporal Oedema in a Child: A Rare Manifestation of Epstein–Barr Virus Infection

**DOI:** 10.1155/crpe/7278571

**Published:** 2025-06-13

**Authors:** Kátia Maurício, Joana De Beir, Rita Alvelos, Inês Sobreira, Joana Santos

**Affiliations:** ^1^Department of Paediatrics, Centro Hospitalar do Baixo Vouga, Aveiro, Portugal; ^2^Department of Paediatrics, Hospital Pediátrico, Unidade Local de Saúde de Coimbra, Coimbra, Portugal

## Abstract

Primary infection by the Epstein–Barr virus (EBV) is common in children, can affect multiple organs and be associated with a wide variety of clinical manifestations. We present the case of a 7-year-old female patient assessed in the emergency department for bitemporal swelling with a one-day evolution, following self-limiting odynophagia and fever 1 week earlier. Physical examination revealed a soft, bitemporal swelling, more evident on the right, painful on palpation, with no other inflammatory signs. The soft tissue ultrasound showed no alterations, and the CT scan showed thickening of the right parietotemporal epicranial soft tissues, of an imprecise nature. At a 2-week follow-up consultation, swelling had completely resolved. The serological study revealed previous contact with cytomegalovirus and positive EBV IgG and IgM with negative EBNA IgG and EA IgG, indicative of acute EBV infection. Bitemporal oedema is a very atypical and rare presentation of primary EBV infection, with very few cases previously reported. The aim of this clinical case is to draw attention to the importance of considering EBV infection in the differential diagnosis of situations like the one described.

**Summary**

• Bitemporal swelling in an otherwise healthy child could be a manifestation of primary EBV infection.
• Other differential diagnosis of acute bitemporal swelling should be excluded.
• Duration of swelling can vary, but it is normally auto limited with complete resolution after a few weeks. Specific treatment is often not needed.

## 1. Introduction

Primary infection by the Epstein–Barr virus (EBV) is a common viral illness in children and adolescents associated with a wide variety of clinical manifestations. Infectious mononucleosis (IM) is the most frequent clinical syndrome encountered during EBV infection and is characterised by a triad of fever, tonsillar pharyngitis and lymphadenopathy [1]. However, EBV can also present with various atypical features that complicate early diagnosis. These include uncommon skin manifestations such as scarlatiniform, vesicular, petechial, or purpuric rashes and rare syndromes such as Gianotti-Crosti and unilateral laterothoracic exanthem [2].

Facial oedema is another atypical sign of EBV infection, most commonly presenting as painless bilateral periorbital swelling, known as Hoagland sign [3]. A more recent prospective study noted Hoagland sign in over half of subjects with acute EBV IM, suggesting it may be more frequent than other atypical signs such as enlarged liver or spleen [4]. Other forms of facial swelling, such as bitemporal oedema, although rare, can occur in the context of EBV infection [5]. This swelling is characterised by a visible enlargement on both sides of the temples, which can be alarming to parents and healthcare providers. The swelling may be due to inflammation of nearby tissue, and it usually resolves with the treatment of the underlying viral infection. However, given its rarity and the potential for other serious underlying causes, bitemporal swelling in a child with EBV infection warrants careful evaluation and management.

## 2. Case Report

A 7-year-old female presented to our emergency department (ED) with bitemporal swelling with a one-day evolution. One week earlier, she referred self-limited odynophagia and fever. The patient denied any history of trauma or contact with chemicals, especially cosmetics, reducing suspicion of a cutaneous hypersensitivity reaction. She denied scalp lesions or having been stung or bitten on the head. There was no reported visual impairment, diplopia, blurred vision, visual field defect, or ophthalmoplegia to suggest ocular involvement.

Physical examination revealed soft, bitemporal swelling, more evident on the right, painful on palpation, with no other inflammatory signs. No signs of odontogenic or oral/pharyngeal inflammation were found. Neurological examinations were unremarkable.

Laboratory studies on admission showed a mild lymphocytosis (5.20 × 10E9/L) with normal leucocyte count (9.4 × 10E9/L). C-reactive protein (CRP) was 1.03 mg/dL (normal < 0.5 mL/dL), and erythrocyte sedimentation rate (ESR) was 31 mm/hour. Haemoglobin (12.0 g/dL), platelets (357 × 10E9/L) and kidney function were normal. She was discharged, medicated with oral antihistamine.

**Figure 1:**
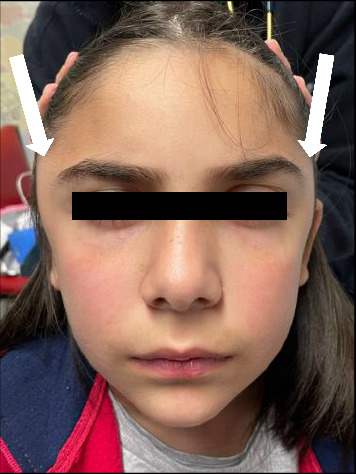
Patient on her second visit to the emergency department. The arrows indicate the temporal swelling, which is more evident on the right-side.

She returned the following day with worsening of the bitemporal swelling (Figure 1) and a single febrile peak. On physical examination, she had limitation of mouth opening due to pain in the temporal region, with no other alterations. Laboratory studies were repeated: ESR 80 mm/h, CRP 2.02 mg/dL and blood smear with reactive lymphocytes, with no other significant changes. Serologies for EBV and cytomegalovirus (CMV) were also taken.

**Figure 2:**
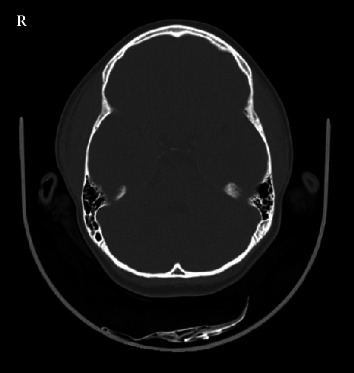
Computed tomography of the head. Axial cut showing thickening of the right parietotemporal epicranial soft tissues.

Computed tomography (CT) of the head showed a thickening of the right parietotemporal epicranial soft tissues of an imprecise nature. No intracranial abnormalities or involvement of the bones was detected (Figure 2). Soft tissue ultrasound was conducted the following morning, as it is unavailable in our hospital during nocturnal hours. The ultrasound of the temporal soft tissue was described as having no alterations. After spending the night in the ED for observation, she was discharged in the morning with ibuprofen and oral antihistamine.

Two weeks later, at the follow-up consultation, the swelling had completely resolved. The serology tests that had been taken earlier revealed previous contact with CMV and were positive for IgG and IgM viral capsid antigen (VCA) of EBV and negative for Epstein–Barr nuclear antigen (EBNA), indicative of acute EBV infection.

## 3. Discussion

Our case highlights a rare association between primary EBV infection and bitemporal oedema in a healthy child—previously reported only once before [5]. Although our patient had mild, self-limiting odynophagia and fever a week earlier, bitemporal oedema was the main presenting feature, highlighting the atypical nature of this presentation. EBV infection was suspected only after a detailed history and confirmed by serology. In children under the age of 10 years, EBV infection often presents atypically or without classic IM features [6]. Unlike Hoagland's sign, which can be an early or sole indicator of acute EBV [1], bitemporal oedema may appear later in the course of disease, as it did in our case and the one described by Friedman et al. [5].

Through the complementary exams conducted, we were able to exclude other differential diagnosis for acute temporal swelling, which include:

1. Osteosarcoma of the temporal bone, albeit rare, can present as an acute swelling. Imaging is essential as these normally demonstrate permeative bone destruction and cortical bone expansion as well as enlargement of both the masseter muscle and medial pterygoid muscle [7].
2. Acute myeloid leukaemia can sometimes present as a progressive bitemporal swelling, normally associated with systemic symptoms such as low-grade fever, anorexia and weight loss [8]. In these cases, blood work with blood smear is important to exclude this diagnosis.
3. Temporal osteitis and myositis as a complication of acute mastoiditis. The infection spreads by contiguity through temporal bone. Temporal swelling can precede clinical appearance of mastoiditis, so postauricular tenderness and protrusion of the auricle may be absent [9]. This diagnosis requires broad spectrum antibiotics and in certain situations surgical drainage.
4. Temporal muscle abscess as a complication of acute otitis media presents with a rapid progression of unilateral temporal swelling in a matter of days [10], unlike the temporal swelling associated with EBV infection which is bilateral.
5. Isolated unilateral temporalis muscle hypertrophy can present as a progressive temporal swelling associated with episodes of recurrent headaches. It is more common in the adult population and is an extremely rare condition in paediatrics, requiring a high degree of suspicion as well as the exclusion of other more common aetiologies of temporal swelling [11].
6. Temporomandibular arthritis needs to be excluded when there is pain with mouth opening. This diagnosis is more common in teenagers with juvenile idiopathic arthritis [12] and is not normally associated with temporal swelling.

None of these aetiologies fit the clinical case presented here, but it is important to exclude other causes of acute bitemporal swelling as some can be potentially life-threatening.

The precise mechanism underlying temporal swelling in the context of EBV infection remains unclear. It has been proposed that direct viral infiltration or a virus-induced inflammatory response within muscle or surrounding soft tissue [13] could potentially be a mechanism. The CT finding of soft tissue thickening in our patient could be consistent with inflammation or oedema of the overlying soft tissues, potentially representing a form of localised inflammation in response to the virus. However, current knowledge is still limited, and further studies exploring the pathophysiology of these phenomena are required.

Management of typical uncomplicated IM is mainly supportive. In our case, the spontaneous resolution of bitemporal swelling and other symptoms within 2 weeks reflects the usual self-limiting course of primary EBV infection [6].

In summary, bitemporal oedema is a rather atypical and rare presentation of primary EBV infection, with no other cases reported in Europe. The aim of this case report is to highlight the importance of considering EBV infection in the differential diagnosis of situations like the one described.

## Data Availability

The data that support the findings of this study are available from the corresponding author upon reasonable request.

## References

[1] Leung A. K. C., Lam J. M., Barankin B. (2024). Infectious Mononucleosis: An Updated Review. *Current Pediatric Reviews*.

[2] Ciccarese G., Trave I., Herzum A., Parodi A., Drago F. (2020). Dermatological Manifestations of Epstein-Barr Virus Systemic Infection: A Case Report and Literature Review. *International Journal of Dermatology*.

[3] Hoagland R. J. (1952). Infectious Mononucleosis. *The American Journal of Medicine*.

[4] Bronz G., Zanetti B. P. E. S. M., Bianchetti M. G. (2023). Bilateral Upper Eyelid Swelling (Hoagland Sign) in Epstein-Barr Infectious Mononucleosis: Prospective Experience. *Infection*.

[5] Friedman N., Fradkin A., Somech R. (2016). The Girl Who Grew Horns: Temporal Swelling as an Atypical Presenting Symptom of Epstein-Barr Virus Infection: Temporal Swelling as an Atypical Presenting Symptom of Epstein-Barr Virus Infection. *The Israel Medical Association Journal*.

[6] Odumade O. A., Hogquist K. A., Balfour H. H. (2011). Progress and Problems in Understanding and Managing Primary Epstein-Barr Virus Infections. *Clinical Microbiology Reviews*.

[7] Villemure-Poliquin N., Trudel M., Labonté S., Blouin V., Fradet G. (2019). Low-Grade Surface Osteosarcoma of the Temporal Bone in Paediatric Patients: A Case Report and Literature Review. *Clinical Medicine Insights: Pediatrics*.

[8] Behari S., Rajput D., Naval R., Yadav K., Tungaria A. (2010). Bilateral Proptosis and Bitemporal Swelling: A Rare Manifestation of Acute Myeloid Leukemia. *Journal of Pediatric Neurosciences*.

[9] Conversano E., Udina C., Cozzi G., Dal Bo S., Marchetti F., Barbi E. (2019). Child With Unilateral Temporal Swelling. *Annals of Emergency Medicine*.

[10] Oki S., Kawabori M., Motegi H. (2019). A Rare Case of Idiopathic Temporal Muscle Abscess in a Nine-Month-Old Infant. *Internal Medicine*.

[11] Ranasinghe J. C., Wickramasinghe C., Rodrigo G. (2018). Isolated Unilateral Temporalis Muscle Hypertrophy in a Child: A Case Report With Literature Review. *BMC Pediatrics*.

[12] Fischer J., Skeie M. S., Rosendahl K. (2020). Prevalence of Temporomandibular Disorder in Children and Adolescents With Juvenile Idiopathic Arthritis—A Norwegian Cross-Sectional Multicentre Study. *BMC Oral Health*.

[13] Sugiyama K., Ito M., Ichimi R. (1997). A Case of Epstein-Barr Virus Infection With Exophthalmos and Ocular Muscle Swelling. *Pediatrics International*.

